# Supplementary material for: Structural atlas of a human gut crassvirus
Source: Nature. 2023 May 3;617(7960):409–16. doi: 10.1038/s41586-023-06019-2 (PMC10172136; doi:10.1038/s41586-023-06019-2)
Supplement: Supplementary file 1 — Reporting Summary [file 41586_2023_6019_MOESM1_ESM.pdf]

nature portfolio

Corresponding author(s): Alfred Antson, Oliver Bayfield

Last updated by author(s): Feb 24, 2023

Reporting Summary

Nature Portfolio wishes to improve the reproducibility of the work that we publish. This form provides structure for consistency and transparency in reporting. For further information on Nature Portfolio policies, see our Editorial Policies and the Editorial Policy Checklist.

Statistics

For all statistical analyses, confirm that the following items are present in the figure legend, table legend, main text, or Methods section.

n/a	Confirmed	
☐	☒	The exact sample size (*n*) for each experimental group/condition, given as a discrete number and unit of measurement
☐	☒	A statement on whether measurements were taken from distinct samples or whether the same sample was measured repeatedly
☒	☐	The statistical test(s) used AND whether they are one- or two-sided<br>*Only common tests should be described solely by name; describe more complex techniques in the Methods section.*
☒	☐	A description of all covariates tested
☒	☐	A description of any assumptions or corrections, such as tests of normality and adjustment for multiple comparisons
☒	☐	A full description of the statistical parameters including central tendency (e.g. means) or other basic estimates (e.g. regression coefficient) AND variation (e.g. standard deviation) or associated estimates of uncertainty (e.g. confidence intervals)
☒	☐	For null hypothesis testing, the test statistic (e.g. *F*, *t*, *r*) with confidence intervals, effect sizes, degrees of freedom and *P* value noted<br>*Give P values as exact values whenever suitable.*
☒	☐	For Bayesian analysis, information on the choice of priors and Markov chain Monte Carlo settings
☒	☐	For hierarchical and complex designs, identification of the appropriate level for tests and full reporting of outcomes
☒	☐	Estimates of effect sizes (e.g. Cohen's *d*, Pearson's *r*), indicating how they were calculated

Our web collection on statistics for biologists contains articles on many of the points above.

Software and code

Policy information about availability of computer code

Data collection	EPU 2.5.0
Data analysis	RELION 3.1; CTFFIND4; Phenix 1.19; Coot 0.9.8.1; ChimeraX 1.5; determine_relative_pixel_size.py 890eb35; TMHMM-2.0; PEAKS Studio 10.5.

For manuscripts utilizing custom algorithms or software that are central to the research but not yet described in published literature, software must be made available to editors and reviewers. We strongly encourage code deposition in a community repository (e.g. GitHub). See the Nature Portfolio guidelines for submitting code & software for further information.

Data

Policy information about availability of data

All manuscripts must include a data availability statement. This statement should provide the following information, where applicable:

- Accession codes, unique identifiers, or web links for publicly available datasets
- A description of any restrictions on data availability
- For clinical datasets or third party data, please ensure that the statement adheres to our policy

Cryo-EM reconstructions and atomic coordinates comprising of the virion capsid and tail structures have been deposited with wwPDB (www.wwpdb.org) under accession codes: EMD-14088, 14089, 14090, 14091, 14092, 14093, 14094, 14100 (maps) and 7QOF, 7QOG, 7QOH, 7QOI, 7QOJ, 7QOK, 7QOL (atomic coordinates), with examples regions of maps and corresponding models in Extended Data Figure 6. Data collection and refinement statistics are presented in Extended Data Table 1. Multiple sequence alignments of ΦcrAss001 virion protein homologues are available at https://ftp.ncbi.nih.gov/pub/yutinn/crAss_structural_prot_2022/.

Human research participants

Policy information about studies involving human research participants and Sex and Gender in Research.

Reporting on sex and gender	N/A as no human research participants.
Population characteristics	N/A as no human research participants.
Recruitment	N/A as no human research participants.
Ethics oversight	N/A as no human research participants.

Note that full information on the approval of the study protocol must also be provided in the manuscript.

Field-specific reporting

Please select the one below that is the best fit for your research. If you are not sure, read the appropriate sections before making your selection.

☒ Life sciences ☐ Behavioural & social sciences ☐ Ecological, evolutionary & environmental sciences

For a reference copy of the document with all sections, see nature.com/documents/nr-reporting-summary-flat.pdf

Life sciences study design

All studies must disclose on these points even when the disclosure is negative.

Sample size	A total of 122,709 images of individual virus particles were used in reconstructions of the virions and sub-assemblies.
Data exclusions	Empty micrographs containing no virus particles were excluded. Images of virus particles were extracted and classified according to cryo-EM data processing procedures in RELION.
Replication	A single sample of purified virus particles derived from bacterial lysate was used to prepare electron microscopy grids. Four cryo-EM data sets of micrographs of virus particles were processed, in total comprising 44,006 micrographs. In addition, 16 negatively stained micrographs were used for initial assessments of sample quality.
Randomization	A bacterial colony or viral plaque was selected at random to initiate propagation of cultures (each colony or plaque contains 1000–10,000 bacteria or viruses). A random subset of virus particles present in solution was embedded in vitreous ice upon freezing.
Blinding	No blinding was performed as the subjects were viruses and bacteria. Cryo-EM data were randomly divided into half-sets and refined independently according to the gold-standard procedure implemented in RELION.

Reporting for specific materials, systems and methods

We require information from authors about some types of materials, experimental systems and methods used in many studies. Here, indicate whether each material, system or method listed is relevant to your study. If you are not sure if a list item applies to your research, read the appropriate section before selecting a response.

Materials & experimental systems

n/a	Involved in the study	
☒	☐	Antibodies
☒	☐	Eukaryotic cell lines
☒	☐	Palaeontology and archaeology
☒	☐	Animals and other organisms
☒	☐	Clinical data
☒	☐	Dual use research of concern

Methods

n/a	Involved in the study	
☒	☐	ChIP-seq
☒	☐	Flow cytometry
☒	☐	MRI-based neuroimaging
